# Wind of Change Challenges Toxicological Regulators

**DOI:** 10.1289/ehp.1104782

**Published:** 2012-08-07

**Authors:** Tewes Tralau, Christian Riebeling, Ralph Pirow, Michael Oelgeschläger, Andrea Seiler, Manfred Liebsch, Andreas Luch

**Affiliations:** German Federal Institute for Risk Assessment (BfR), Center for Alternative Methods to Animal Experiments (ZEBET), Berlin, Germany

**Keywords:** alternative testing approach, chemical safety testing, *in vitro* testing strategy, *in vivo* testing, regulatory acceptance, stem cells, Tox-Test Dummy

## Abstract

Background: In biomedical research, the past two decades have seen the advent of *in vitro* model systems based on stem cells, humanized cell lines, and engineered organotypic tissues, as well as numerous cellular assays based on primarily established tumor-derived cell lines and their genetically modified derivatives.

Objective: There are high hopes that these systems might replace the need for animal testing in regulatory toxicology. However, despite increasing pressure in recent years to reduce animal testing, regulators are still reluctant to adopt *in vitro* approaches on a large scale. It thus seems appropriate to consider how we could realistically perform regulatory toxicity testing using *in vitro* assays only.

Discussion and Conclusion: Here, we suggest an *in vitro*–only approach for regulatory testing that will benefit consumers, industry, and regulators alike.

Since the 1980s, there has been a remarkable surge in biomedical and pharmaceutical research and in the development of new chemicals and marketed commodities (Binetti et al. 2008). Although these products have contributed to consumer well-being and economic success, regulators, in response to increasing concerns about environmental and consumer safety, have introduced new safety regulations and testing requirements (e.g., Makris et al. 2009).

Historically, due in part to the absence of non-animal alternatives, toxicological safety testing has emphasized the use of animal-based studies (Schardein and Keller, 1989). However, such tests are ethically arguable and can be expensive and time consuming, particularly with regard to tests requiring a prolonged exposure or the breeding of multiple generations. With regard to cost, it still remains to be demonstrated if alternative testing methods will always be an improvement. Currently, the main promise is that the use of human-derived cells and tissues will increase the predictivity toward human toxicological effects (Adler et al. 2011; Wobus and Löser 2011). Thus, there is increasing pressure from industry and the public to reduce animal tests wherever possible and eventually to abandon them altogether. Initially, most *in vitro* tests were relatively simple biochemical or cellular assays, limiting their applicability (Laschinski et al. 1991). During the last decade, however, a number of major advances have been made in molecular toxicology, and today’s portfolio of non-animal methods is far more versatile (Adler et al. 2011; Wobus and Löser 2011). Examples include the development of novel stem cell techniques, organotypic three-dimensional (3D) cell models, *in vitro* disease models, and an increasing number of *in vitro* cell-based “omics” and *in silico* methods (reviewed by Adler et al. 2011; Wobus and Löser 2011).

The pharmaceutical industry has adapted many of these methods in order to screen large substance libraries in an effort to minimize attrition rates, while chemical industry and regulators alike are still evaluating the use of these new technologies for toxicological risk assessment (Pampaloni and Stelzer 2010). After years of debate, the first *in vitro* assays for local acute toxicity have now been accepted internationally as testing guidelines of the Organisation for Economic Co-operation and Development (OECD) (reviewed by Liebsch et al. 2011). There are good reasons for this rather slow and cautious approach by toxicological regulators who have to treat safety as paramount and hence want a standardized and validated set of testing methods accepted and adhered to internationally. Regulatory acceptance of *in vitro* approaches is especially difficult when applied to the systemic toxicological end points of repeated dose toxicity, carcinogenicity, and reproductive toxicity, where considerations have to include low dose levels and chronic exposures. The corresponding adverse effects are rarely limited to a single organ, but instead tend to be diverse and can encompass the whole organism. Moreover, regulatory toxicity testing faces the problem of possible false-negative testing. False negatives are a serious concern for toxicological test systems, but such results may well be acceptable during pharmaceutical screens. However, significant levels of research funding for regulatory toxicology have been lacking, and thus the field has depended generally on data generated in studies of limited scope. For example, the development of organotypic toxicity models has depended largely on investigations in medical tissue engineering and tissue regeneration, currently one of the major medical research areas. Meanwhile, the political pressure to implement testing alternatives for regulatory toxicology has increased greatly as demonstrated by the release of the National Research Council’s (NRC) vision and strategy for toxicity testing in the 21st century (NRC 2007), the seventh amendment of the European Cosmetics Directive in 2003 (European Commission 1976, 2003) and the REACH (Registration, Evaluation, Authorisation and Restriction of Chemicals) program of 2006 (European Commission 2006). What was once a breeze has now become a gale that will ultimately force regulators to be more proactive in incorporating non-animal alternatives into the regulatory decision-making process. Therefore, it is appropriate at this time to discuss the criteria that a prospective *in vitro*–only testing scheme will need to meet in order to satisfy regulatory requirements and to outline how these criteria can be achieved.

## Current Efforts

The NRC’s 2007 vision of moving toxicity testing away from animal testing to *in vitro* and, ultimately to *in silico* approaches, resulted in the launching of several projects. Most notable is a formal collaborative effort, known as “Tox21” (Toxicology Testing in the 21st Century), by various U.S. agencies, including the Chemical Genomics Center of the National Institutes of Health, the National Toxicology Program, the U.S. Environmental Protection Agency (EPA), the National Center for Computational Toxicology, and more recently, the Food and Drug Administration (FDA), to screen a large number of chemicals of environmental concern across several hundred high-throughput *in vitro* methods. The aim is to identify substances of greatest concern and also to evaluate the performance of each individual test method singly or in combination with other assays (Collins et al. 2008; Krewski et al. 2010). All data are computationally processed and analyzed for toxicologically relevant cellular pathways, which ultimately could lead to *in silico* prediction models such as those envisioned by the U.S. EPA’s ExpoCast, virtual liver (v-Liver™), and virtual embryo (v-Embryo™) projects (Kavlock and Dix 2010). These projects aim to provide a toxicological assessment of the currently existing chemical space, including numerous untested legacy chemicals, and to prioritize animal testing (Dix et al. 2007; Judson et al. 2010). Moreover, the National Center for Advancing Translational Science (NCATS) together with the Defense Advanced Research Projects Agency (DARPA) and the FDA have committed 140 million U.S. dollars over 5 years to design a tissue “chip” for drug screening (DARPA 2011; NCATS 2011). The Japan HPV (high production volume) Challenge Program of 2007 addresses a similar challenge (Ministry of Economy, Trade and Industry, Ministry of the Environment of the Japanese Government, Ministry of Health, Labor and Welfare 2007) and, in the European Union, the AXLR8 (pronounced “accelerate”) project is a collaborative initiative for developing alternative methods for chemical testing (AXLR8 Consortium 2010). Thus, at this time, it is appropriate to ask what types of data will be expected by the regulatory authorities from the chemical industry in the future when a new product is proposed for commercial use. These efforts will undoubtedly contribute significantly to the answer. A high-throughput screening approach will be used most likely during the research and development of a product. However, it might not be well suited enough for the regulatory assessment of a single product. For regulatory acceptance, we suggest starting the development of a directed approach comprising a set of integrated assays that can be offered by contract laboratories in the same way that animal testing is conducted currently. This approach is in the interest of the regulators who then will be able to deal with a manageable data set—the evaluation of which would not require proficiency in a bewildering number of alternative testing systems. Moreover, it would provide industry with a defined and clear financial framework while also offering advantages to all stakeholders.

## Calculating the Odds—Involuntary Heroism of Animals

A toxicological risk assessment considers all of the available toxicological data for a given substance, taking into account the likelihood of exposure and the routes, magnitude, and duration of exposure, and finally reaches a conclusion based on the doses the average person is exposed to. This approach of an exposure-based risk assessment is applied to substances already on the market, as well as newly developed products, and is necessarily based on many assumptions and extrapolations. The assessment of actual or expected exposure typically relies on mathematical modeling and requires as much reliable data as possible in order to keep uncertainties to a minimum. Data on exposure can ideally be extrapolated from similar products or are available from epidemiological data, biomonitoring programs, and well-conducted surveys. Meanwhile, toxicological data originate predominantly from animal studies. From a risk assessor’s point of view, the highest risk is not posed by acutely toxic chemicals but by those that show systemic, reproductive, developmental, and carcinogenic effects under long-term exposure conditions, including low doses. Therefore, animal studies required for the regulatory acceptance of high-volume production chemicals include, among others, the so-called two-generation study (OECD 2001). This study is used to evaluate the ability of a chemical to induce developmental, reproductive, or chronic effects and poses the main burden in terms of animal usage and costs. For a single chemical, a two-generation study uses on average 3,200 animals (normally rats) (Rovida and Hartung 2009) and costs close to half a million U.S. dollars for one species (Fleischer 2007; U.S. EPA 1998). Testing a second mammalian species, as is sometimes recommended, doubles the price tag. In addition, it can take up to half a year to gather the necessary data and prepare the proper dossier for submission to regulatory agencies. The two-generation study could be replaced in the near future by an extended one-generation reproductive toxicity study (EOGRTS) (OECD 2011); however, its full legal implementation is pending. In an effort to reduce animal numbers, the EOGRTS would monitor more parameters and routinely omit the second generation, thus requiring 40–60% fewer animals. Nevertheless, the long and intense debate surrounding the EOGRTS, still an *in vivo* method, perfectly illustrates the rift between public expectation and the regulatory demand for validated and tested methods. Ironically animal models as such have never been validated; rather, it has been taken on good faith that these studies are the best approach for protecting humans. Bcause of its universality, this approach has mostly stood the test of time and shaped our understanding of toxicological adversity. Now this universality poses a problem as no single *in vitro* test system can offer the same degree of complexity.

The two-generation study requires the performance of daily clinical observations, including the examination of parental body weight and feeding behavior, as well as of the female estrous cycle and male sperm parameters. Physical development of the offspring is monitored by body weight gain and other parameters (e.g., ear and eye opening, tooth eruption, hair growth). Further examinations include gross necropsy, organ weight, and histopathology of the following organs: vagina, uterus with cervix and ovaries, testis, epididymis, seminal vesicles, prostate, and coagulating gland, as well as grossly abnormal tissue and target organs from all pups with external abnormalities or clinical signs. In addition, follow-up studies such as a developmental neurotoxicity study (OECD 2007) might be necessary if, for instance, the investigator is alert to behavioral abnormalities.

## Challenging High Expectations

All of this information, and ideally even more, would have to be obtained from a battery of *in vitro* tests. Further, such a battery would ideally have a predictivity for human health effects equal to or better than the typical 60% seen with animal studies (reviewed by Hartung 2009). This rather low predictivity is mainly due to differences in species-specific pharmacokinetics and xenobiotic metabolism, which are two inherent limitations of *in vivo* systems. This is sadly exemplified by the thalidomide saga, one of the most dramatic failures of animal model testing (Gilbert 2003).

**Figure 1:**
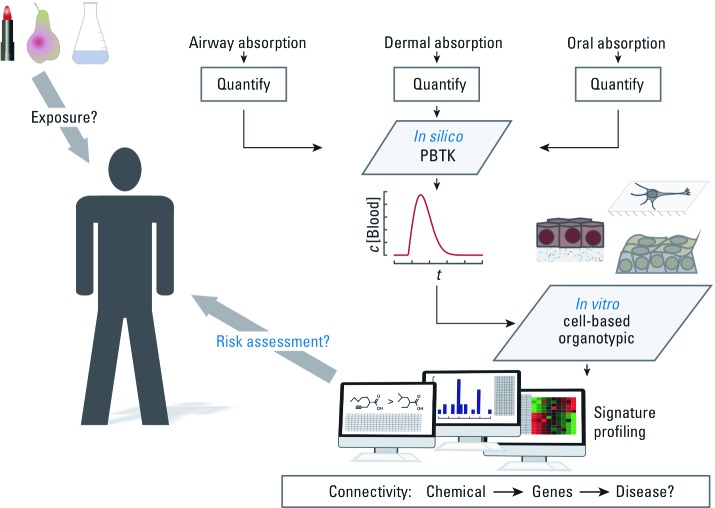
Schematic overview of the required components of a Tox-Test Dummy. Abbreviations: *c*[Blood], concentration of a substance in blood; *t*, time.

However, the metabolic barrier does not necessarily apply to *in vitro* tests, as cell-based systems can be either of human origin or be humanized with regard to key metabolic enzymes. This elimination of the species barrier is potentially the biggest advantage of *in vitro* systems and should increase predictivity. However, a humanized *in vitro* testing scheme, or “Tox-Test Dummy” (Figure 1), will face a different dilemma: specifically, a limited data set for validation. Human data usually originate from accidents, individual case reports, or retrospective studies. Using existing *in vivo* animal data for comparison will not necessarily solve this problem. Out of several thousand developmental toxicants identified in animal studies, only about 50 have been reported to exhibit embryotoxic effects in humans (Schardein and Keller 1989). A solution to this problem could lie in the analysis and comparison of adversely affected biochemical pathways in humans and animals, respectively. The ToxCast program, as part of Tox21, currently identifies biological pathways that are altered as a consequence of toxicological insult (Chandler et al. 2011; Kleinstreuer et al. 2011) Linked with other available *in vitro* and *in vivo* data from human exposure and animal testing, these data can be used not only to improve understanding of the underlying biochemistry, but also to elucidate differences and similarities between species. Ultimately, such a detailed understanding of the affected pathways across species might be used to validate the human relevance of *in vitro* assays. However, we believe that regulators at a certain, not too distant, point (as discussed below) will accept the inherent limitations of *in vitro* testing in the same way as they currently accept the limitations of *in vivo* testing.

*In vitro* testing batteries are by design deconstructive, which makes it difficult to determine which organs a substance will potentially affect given realistic external exposures, and what target dose level (or levels) should be considered. In the absence of an intact organism, this issue is far from trivial. A previously untested chemical might be distributed throughout an organism and have more than one target. In addition, the potential targets might be mechanistically unrelated.

So, do we have to rebuild and treat all of the > 40 human organs or of the > 400 cell types? The answer is yes and no. To limit testing to truly relevant targets, we have to assess all routes of exposure and estimate the corresponding doses for potential target organs or tissues after external exposure. Typical routes of exposure are dermal, inhalation, and ingestion. Absorption through the mucous membranes can be modeled *in silico*. Likewise, there are validated methods to measure dermal absorption *in vitro*, and cell systems for other barriers are available as well (Adler et al. 2011; European Commission 2008). If absorbed, the chemical is likely to reach the bloodstream. Using physiologically based toxicokinetic (PBTK) modeling, it is then possible to predict organ exposure levels and thereby establish relevant concentrations for any subsequent *in vitro* testing (Figure 1) (Mielke et al. 2011). The potential of coupling organ-specific PBTK to downstream assays such as gene arrays was recently demonstrated by Meyer et al. (2012) who used this approach to investigate the *in vivo* activity of pravastatin.

One of the most important organs will undoubtedly be the liver, where phase I metabolism facilitates excretion but also increases the toxicity of some substances. Hence, liver metabolism is currently one of the most important research areas of *in vitro* testing. Although current high-throughput projects such as ToxCast typically do not include xenobiotic metabolism in their *in vitro* assays, they recognize that this issue is critical to the success of their efforts. Currently available systems for mimicking liver metabolism include the use of liver homogenate (S9 extracts), transgenic cell lines, hepatocyte-like cell monolayers, and 3D organotypic cultures (Adler et al. 2011; Esch et al. 2011; Giri et al. 2011; Landsiedel et al. 2011). The S9 extracts are frequently sourced from rodents because of the limited availability of pathologically unaffected human liver tissue or primary human hepatocytes, which raises concerns about species specificity. However, the generation of hepatocytes from induced pluripotent stem cells may give rise to an unlimited resource of human material (Chen et al. 2012; Medine et al. 2010; Takayama et al. 2012; Tralau and Luch 2012; Wongkajornsilp et al. 2012). We will not discuss the individual pros and cons of these systems here, but we will assume that such systems will be integrated into routine *in vitro* testing. One of the most important issues for an integrated model is how the resulting metabolites are transferred to the next assay. The use of S9 extracts has proven itself to be problematic in cell culture, and likewise the supernatant of liver-cell culture (Hettwer et al. 2010). At the moment, these issues are going to be addressed, for example, by the coupling of assays via suitable metabolically competent organotypic cultures (Sonntag et al. 2010; Sung and Shuler 2010).

In the next step, the chemical and its metabolites (if any) need to be tested for their tissue-barrier mobility. In the case of impermeable substances, the exclusion of whole organs or tissues from testing will help to minimize the need for *in vitro* testing and avoid false-positive results. Again, the application of PBTK modeling allows for the prediction of realistic concentrations and doses and helps to prioritize subsequent testing. When metabolism is known, PBTK modeling can even model the homeostasis of whole organs (Subramanian et al. 2008). Likewise, simple assays with false-negative rates close to zero could be used to prioritize chemicals for more involved organotypic assays, even if the initial screening assays have high false-positive rates.

If the primary target of chronic exposure and toxicity is the liver, the second most prevalent target is the kidneys, followed by the reproductive organs, the brain, hematopoietic tissues, and bone. Cell culture models are available for most of these organs, either as immortal cell lines, primary cultures, reprogrammed stem cells, or even organotypic cultures (i.e., Peljto and Wichterle 2011; Wobus and Löser 2011). For other organs, miniaturized chips, such as the “lung on a chip” (Huh et al. 2010), can be used to measure cellular reactions under physiological conditions. All of these systems allow the detection of necrotic and apoptotic cell death. Nevertheless, a major challenge is the reliable detection of carcinogenic and mutagenic events as well as developmental defects. Many of the underlying molecular pathways are known and each year we learn more about the respective key molecules. For many of these pathways, biochemical and cellular assays are available, as are reporter cell lines (reviewed by Schenk et al. 2010). A reliable combined molecular testing strategy, however, is usually missing, because we still do not understand the key events well enough. At the same time, it appears that “omics” approaches can be used to identify toxic signature patterns within cellular metabolic pathways *in vitro* (Winkler et al. 2009). Such an approach would not only increase predictivity and be suitable for high-throughput screening, but would also allow simultaneous measurement of multiple end points.

What are the challenges of an *in vitro* approach, and what performance can we realistically expect? We will try to elucidate these questions using the example of a putative herbicide that will turn out to be a neurotoxicant after metabolic conversion. First, a skin-barrier model would be used to determine the amount of herbicide that reaches the blood after dermal exposure. Next, a model for the toxicant’s distribution in blood, such as a PBTK model, would be used to estimate the concentration reaching the liver. The liver model must then be used to metabolize the agent, and the resulting metabolite (or metabolites) would be applied to a set of organ-mimicking *in vitro* systems, including a model of the blood–brain barrier. Finally, a brain model would be exposed to the molecules that are capable of crossing the blood–brain barrier. For the sake of our example, we assume that only the brain model shows an adverse response, and that parallel assays (e.g., assays for liver and spleen toxicity) do not contribute to the detection of our assumed neurotoxin. For good predictivity, we also assumed that all models are composed of nearly all cell types representing the modeled organ, for instance, by appropriate differentiation of human induced pluripotent stem cells. Altogether, five modeled steps would be required for hazard identification. If we assign a worst-case predictivity of 75% to each step, the total predictivity would be about 24%. To reach the 60% predictivity of animal models toward human toxicants, each *in vitro* step would have to exhibit > 90% predictivity, and to achieve 95% overall predictivity, each individual assay would have to perform better than 99%. Parallel assays in other organ models that are not mentioned here would not affect this calculation. However, they would have to exhibit the same predictivity to yield a similar overall predictivity in different scenarios.

At first analysis, these requirements look like a formidable challenge. However, in combination with metabolomics and transcriptomics approaches, current *in vitro* models already tend to reach 80–95% predictivity, sometimes even more. Gene arrays have already been used to predict liver damage in primary rat hepatocytes with 91% sensitivity and 88% specificity (Dai et al. 2006). Similarly, a recent proof-of-concept study used transcriptomic analysis to identify chemical carcinogens in hepatocyte-like cells derived from human embryonic stem cells. The overall accuracy of this system came close to 96% (Yildirimman et al. 2011). Metabolomic analysis of human WA09 embryonic stem cells identified teratogenic substances, including thalidomide, with 88% predictivity (Kleinstreuer et al. 2011). Likewise the combination of read-across with several quantitative structure–activity relationship (QSAR) models allowed Hewitt et al. (2010) to reach 89% predictivity for developmental toxicity. Even for the notorious non-genotoxic carcinogens, toxicogenomic approaches reach a predictivity of up to 80%, which is superior to the classic rodent–cancer bioassay (Fielden et al. 2011; Liu et al. 2011; Low et al. 2011). Thus, for our suggested Tox-Test Dummy, it seems realistic to expect an overall predictivity of 51–86% based on current assays, although predictivity would be higher for common scenarios that do not involve neurotoxicity, and therefore would require only four steps. Moreover, this calculation does not account for the additional benefits that would result from the use of human cells and the integration of several organ models onto a single chip (Esch et al. 2011; Huh et al. 2010). In a recent proof-of-principle study, Prot et al. (2012) recapitulated major aspects of acetaminophen hepatotoxicity on a biochip.

At some stages of the Tox-Test Dummy, non-human cells might be as predictive as human cells and may even be more useful. Effects on the developing fetus can be assessed using stem cells, such as the mouse embryonic stem-cell test (EST) (Seiler and Spielmann 2011). This test has already been validated by the European Center for the Validation of Alternative Methods (Genschow et al. 2004). Species-specific metabolism is the predominant, if not the sole, reason for the poor transferability of data between species for developmental toxicants (Dorne 2010; Voisin et al. 1990; Walton et al. 2001). The addition of maternal metabolites (e.g., those from the aforementioned incubation with a human hepatocyte cell-like system or an S9 extract of the latter) together with a model of the placental barrier will largely eliminate this shortcoming. Using mouse stem cells in the subsequent differentiation assay would thus be justifiable, and even preferable, because differentiation protocols are much shorter in mouse stem cells compared with human cells. With the addition of end points for developmental neurotoxicity and osteotoxicity (two other versions of the EST that are currently in development) this test promises to surpass the predictivity of corresponding animal models (e.g., Zimmer et al. 2011; zur Nieden et al. 2010). Naturally, as part of good scientific practice, and similar to animal testing in a second species, a parallel unrelated assay that addresses the same question should be performed. In the case of embryotoxicity, one could imagine complementing the envisioned metabolically competent version of the EST by testing the identified metabolites on zebrafish or hen eggs. Comparing the results would either boost confidence or trigger a second look at the chemical’s action.

Generally speaking, embryonic stem cells and induced pluripotent stem cells clearly are promising in that they have the potency to differentiate into nearly every cell type—even organ-like structures. However, it is important to confirm that the resulting structures are representative of adult tissue. When this is not the case, adult-derived differentiated (non-stem) cell lines, which also have a more stable phenotype, may be a better choice (Pannetier and Feil 2007; Wobus and Löser 2011; Zeng and Rao 2006).

## Outlook

Combining these assays into a molecular Tox-Test Dummy would cover many toxicological aspects currently assayed *in vivo*. Unarguably, many of these systems still need further development such as the establishment of reliable “omics” signatures, the refinement of organotypic cell cultures, and cellular differentiation protocols. Most important, how to integrate these systems into a practical testing strategy such as a Tox-Test Dummy has been hardly addressed so far. Nevertheless, these are issues that can and will be solved in time. The ultimate demand of organotypic systems for all tissues seems now less utopian than it was 10 years ago.

**Figure 2:**
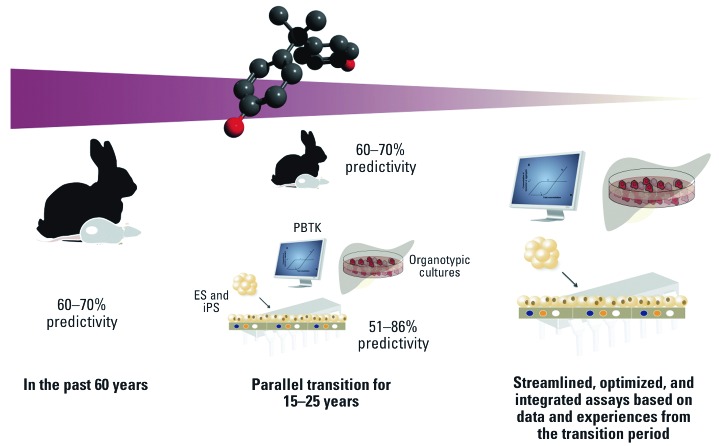
Introduction of *in vitro* testing in parallel to current regulatory *in vivo* tests would allow for the optimization and targeted development of an integrated testing strategy without compromising safety. Practical experiences will either increase trust in the systems used or highlight weaknesses to be addressed. Eventually such an integrated approach will have a higher predictivity than current systems, which would provide the opportunity to phase out live-animal testing. Abbreviations: ES, embryonic stem cell; iPS, induced pluripotent stem cells.

The two most quoted arguments against an *in vitro* testing battery are concerns about the relevance of any observed effects and the issue of validation. Although the latter is a valid point from a health and safety perspective, it is a logic paradox as animal testing itself has never been validated. However, *in vitro* validation studies are necessarily based on data generated by these unvalidated methods. We argue that regulators will have to accept some of the limitations surrounding the issue of validation, which may seem radical. However, we are not suggesting that validation trials be abandoned. In fact, validation could be improved by incorporating increasing knowledge about biological pathways with toxicologal relevance. Rather, we propose that a Tox-Test Dummy is being initially regarded as equivalent to testing in a second species. Its use alongside established *in vivo* tests would allow for the new concept to be evaluated while maintaining a maximal degree of safety (Figure 2). Meanwhile, the suggested combined use of an exposure-based target dose and metabolite assessment would ensure that test systems are exposed to relevant doses. In its idealization such a Tox-Test Dummy would not only cover most regulatory testing needs, but it also would be quicker and more likely to have a better predictivity than the current system. Ideally, the economics of going *in vitro* should be enticing as well, or, at the very least, on par with the cost and time of animal testing. A look at the ever declining price tags for “omics” technologies shows us that this is a realistic expectation.

Money cannot solve everything, but increased funding efforts by the authorities and industry will definitely move us toward the Tox-Test Dummy more quickly. The efforts have to go directly into an integrated, multidisciplinary approach, bringing together the available assays, rather than continuing scattered funding of specialized areas. Further, we recommend developing performance standards for such a prospective Tox-Test Dummy rather than specifying assays. This approach would open development of the Tox-Test Dummy to every possibility and let the best concept(s) win. Finally, given the ethical debate surrounding the use of human stem cells, we encourage the toxicological community to engage with lawmakers to help them develop a better understanding and more realistic perspective on these issues. Arguments about concepts are a necessary process of any scientific and political debate. However, for regulatory toxicology the ongoing debate needs to be more focused or the gale will grow into a storm.
